# A decrease in serum dihydrotestosterone levels in 9-year-old Vietnamese children from a dioxin exposure area

**DOI:** 10.1265/ehpm.24-00190

**Published:** 2024-10-26

**Authors:** Oanh Thi Phuong Nguyen, Seijiro Honma, Phuc Duc Hoang, Khanh Van Nguyen, Anh Thai Le, Shoji F. Nakayama, Manh Dung Ho, Viet Hoang Nguyen, Tung Van Dao, Nhu Duc Dang, Tan Thi Minh Ngo, Thuc Van Pham, Toan Van Ngo, Chi Van Vo, Hideaki Nakagawa, Teruhiko Kido

**Affiliations:** 1 School of Preventive Medicine and Public Health, Hanoi Medical University, Hanoi, Viet Nam; 2 Faculty of Health Sciences, Institute of Medical, Pharmaceutical and Health Sciences, Kanazawa University, Kanazawa, Japan; 3 Hanoi Department of Health, Hanoi, Viet Nam; 4 University of Medicine and Pharmacy, Vietnam National University, Hanoi, Viet Nam; 5 IDEA Consultants, Inc, Tokyo, Japan; 6 Centre for Health and Environmental Risk Research, National Institute, Tsukuba, Japan; 7 Lac Hong University, Dong Nai, Viet Nam; 8 Hanoi Medical University, Hanoi, Viet Nam; 9 Hai Phong Medical College, Hai Phong, Viet Nam; 10 Phu Cat Health Centre, Binh Dinh, Viet Nam; 11 Kanazawa Medical University, Kanazawa, Japan

**Keywords:** Dihydrotestosterone, Testosterone, Androgen disruption, Dioxin exposure, 9-year-old children, Vietnamese cohort

## Abstract

**Background:**

Dioxin is an environmental pollutant as well as an endocrine disruptor in humans. Our longitudinal study wants to clarify the relationship between dioxin exposure and endocrine disorders in children living in the Vietnamese dioxin hotspot.

**Method:**

Seventeen congeners of polychlorinated dibenzo-p-dioxins/polychlorinated dibenzo-furans (PCDDs/PCDFs) in maternal breast milk and seven serum steroid hormones in children of 43 and 46 mothers and their 9-year-old children from the non-exposure and the hotspot areas were measured, respectively. The steroid metabolic enzyme ratios were calculated based on the hormone level ratio.

**Results:**

Most dioxin/furan congeners and toxic equivalents (TEQs) levels were significantly higher in the hotspot than in the non-exposure area, except for 2,4,7,8-TeCDF. The height and weight of girls from the hotspot area were substantially lower and inversely correlated with dioxin congener levels/total TEQs level dioxin. The dihydrotestosterone (DHT) levels in the hotspot were markedly lower than those in non-exposed in both genders. The cortisol concentrations were significantly higher in the hotspot than those from the non-exposure area only in the girls. The DHT/testosterone ratios that exhibited the 5α- or 5β-reductase activity declined by 50% in the hotspot area for both genders. The DHT levels showed strong inverse correlations with almost the PCDDs/PCDFs congeners and total TEQs dioxin in breast milk.

**Conclusions:**

This finding suggests that dioxin exposure in maternal breast milk might impact children’s endocrine system until 9 years old, especially on the DHT biosynthesis.

## Graphical Abstract

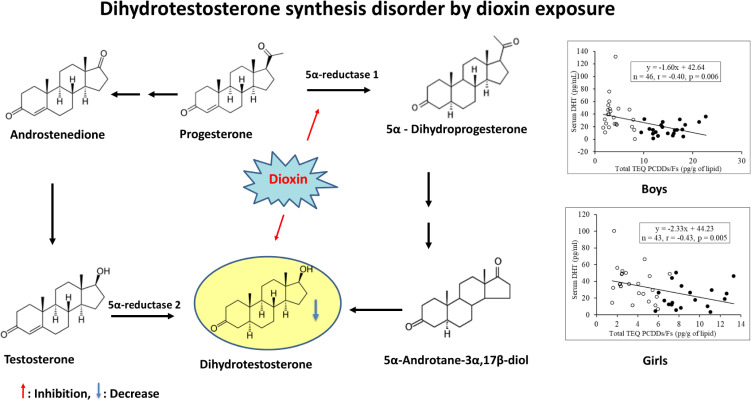

## Introduction

Dioxin is a lipophilic and bioaccumulating environmental pollutant in humans and is known as an endocrine disruptor. Among the dioxin congeners, 2,3,7,8-tetrachlorodibenzo-p-dioxin (TCDD) is the most toxic chemical compound and has a biological half-life of 7–10 years [1]. When defoliant agents including dioxin were sprayed in southern Vietnam during the Vietnam War (1965–1971), it caused extensive health damage to many people. Its health damages were reported to cause diseases such as peripheral neuropathy, several cancers, skin disorders, teratogenicity, chloracne, etc [2].

The steroid hormone plays an important mediating role in brain development and the growth of the reproductive organs [3]. Therefore, since 2008, we have started to research the relationship between health damage and hormone disruptions in children and adult humans in the hotspot areas. To determine the extent to which hormone disruption takes place in the endocrine organ, we estimated several steroid hormones and their intermediates using liquid chromatography-tandem mass spectrometry (LC-MS/MS) in children from the dioxin-exposed area in Vietnam.

In previous studies, we found the characteristics of age by the stage of generation. In the first generation (48–79 years old), we observed an increase in the serum androgen (testosterone (T), dihydrotestosterone (DHT)) and estradiol with age and those were reported to be a risk factor for prostate cancer [4]. In the second generation (25–35 years old), the delivery mothers, a relationship between low birthweight and an increase in cortisol in mothers was found by Tung et al [5]. In the third generation, the children, the dehydroepiandrosterone (DHEA) and T levels declined in follow-up studies at 3 to 7 years old [6–9]. Many researchers reported that higher dioxin levels in breast milk and higher prenatal dioxin levels were associated with the feminized play in boys and affected neurodevelopment involving autistic traits in boys [10–13]. Furthermore, a Russian children study reported that dioxin produced a hormone disorder by T decline and reduced the capacity of testis and sperm level and activity on pubertal timing (17–18 years) in Burns et al and Minguez-Alarcon et al [14, 15].

Our previous studies showed that the decrease in DHEA might be primarily due to the suppression of the CYP17,20-Lyase activity, and the reduction in T was due to the suppression of the 17β-HSD activity by dioxin [7, 8]. Therefore, we conducted this research involving 9-year-old children to investigate the effect of dioxin on serum sex steroid hormones having a strong biological activity, especially DHT and estradiol using LC-MS/MS. We also effort to explain the mechanism of hormone disruption resulting from dioxin.

## Material and method

### Study area

The dioxin-contaminated area chosen in the current study was the area surrounding Phu Cat Airbase, located in the south of Vietnam, which has been well-known as one of the three Vietnam dioxin hotspots [16]. Kim Bang District, Ha Nam province, which was not sprayed with dioxin during the Vietnam War, has been chosen as the non-exposure area in our studies [17].

### Subjects and sampling

**Fig. 1:**
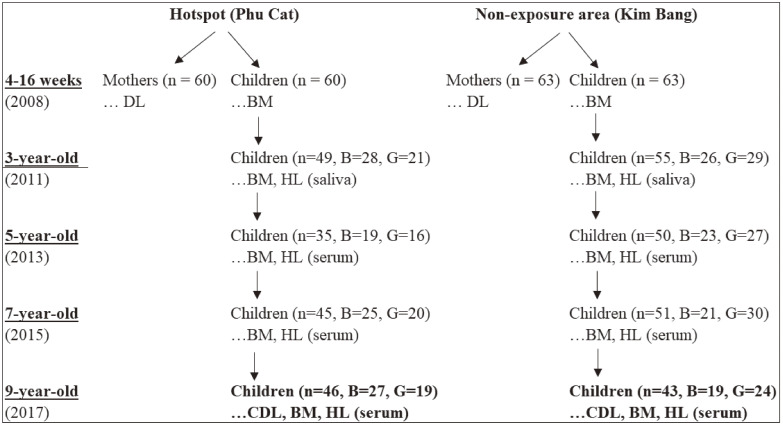
The outline of this research *Note:* B: Boys, G: Girls, BM: Body measurement, HL: Hormone levels, CDL: Children dioxin level in serum, DL: Dioxin levels in maternal milk

Starting the initial study in 2008 with the participation of lactating mothers aged 20–30 years and their infants aged 7–16 weeks (60 mother-child pairs in the hotspot and 63 in the non-exposure area), we examined children for each two years from 2011 [6–8]. Their characteristics were described previously [7, 21]. Due to drop by traveling, visiting relatives, illness, or parent refusal, follow-up 9-year-old children who participated in the 2017 sample collection were 46 and 43 child-mother pairs in the hotspot and the non-exposure area, respectively. The concentration of maternal dioxin was collected from our database of dioxin in the mother’s breastmilk from both areas. On the other hand, blood samples were collected from children by the local medical staff. The blood was then centrifugated and serum was immediately obtained. The subject of this follow-up study is shown in Fig. 1. Body measurements of children (height, weight, head and chest circumferences) were also obtained.

### Analysis of serum steroid hormones using liquid chromatography – tandem mass spectrometry (LC-MS/MS)

LC-MS/MS was performed using the Shimadzu 8060 triple-stage quadrupole mass spectrometer equipped with a positive electrospray ionization (ESI) source (Shimadzu, Kyoto, Japan) and a Shimadzu HPLC system (LC-30AD pump, SIL-30AB autosampler, LC-20AB prominence liquid chromatography, CTO-20A column oven, Shimadzu, Kyoto, Japan). The column was a Kinetex C18 (1.7 µm, 2.1x150 mm, Phenomenex, USA) used at 50 °C. The mobile phase consisting of 0.1% formic acid (A) and acetonitrile (B) was used with gradient elution of A/B = 50 to 100 at the flow rate of 0.45 mL/min. Hormone estimation using LC-MS/MS was carried out at the National Environmental Inst. in Japan.

The hormone determination outline was confirmed in the previous method [7]. Serum (50–200 µL) was diluted to 1 mL with purified water, and 100 µL of a methanol solution containing an internal standard (IS) mixture (cortisol-^2^H_4_; 1000 pg; androstenedione-^13^C_3_: 200 pg (A-dione); T-^2^H_5_, DHT-^2^H_3_, DHEA-^2^H_6_, estrone-^13^C_3_, and estradiol-^2^H_4_: 100 pg) was added to the serum solution. The stable isotope labeled compounds used as the IS were as follows: cortisol-9,11,12,12-^2^H_4_, A-dione-2,3,4-^13^C_3_, T-2,2,4,6,6-^2^H_5_, DHT-16,16,17-^2^H_3_, DHEA-2,2,3,4,6,6-^2^H_6_, estrone-2,3,4-^13^C_3_, and estradiol-2,4,16,16-^2^H_4_. After extraction with ethyl acetate according to the previously reported method, the extract was applied onto a reversed-phase solid cartridge column (C18, 60 mg/3 mL) to remove any impurities [22]. The obtained product was converted to a picolinic acid derivative in tetrahydrofuran using the picolinic acid anhydride [18]. The picolinic acid derivative was purified by a silica gel cartridge column (500 mg/3 mL) to remove excess reagents. After evaporation, the purified derivative was dissolved in 100 µL of 60% acetonitrile/0.1% formic acid solution, and 10 µL of this solution was injected into the LC-MS/MS (Shimadzu 8060 triple). The column used for the analysis was a Kinetex (C18, 1.8 µm, 2.1 × 100 mm), and the elution solvent was a stepwise concentration gradient using a 0.1% formic acid aqueous solution and acetonitrile. The measuring ions (m/z) for the picolinic acid derivatives (PA) or non-derivatized steroids are as follows: cortisol-PA and cortisol-PA-^2^H_4_, 468.2/267.2 and 472/313.2; DHEA-PA and DHEA-PA-^2^H_6_, 394.1/175.1 and 400.2/259.2; A-dione and A-dione-^13^C_3_, 287.1/97.1 and 290.1/100.1; T-PA and T-PA-^2^H_5_, 394.1/253.2 and 399.1/258.2; DHT-PA and DHT-PA-^2^H_3_, 396.1/255.2 and 399.1/206.2; estrone-PA and estrone-PA-^13^C_3_, 376.1/157.1 and 379/160.1; estradiol-2PA and estradiol-2PA-^2^H_4_, 483.1/264.1 and 487.0/266.1. The quantitative value was obtained from each calibration curve and converted into the concentration per serum. The lower limits of quantitation for cortisol-PA, DHEA-PA, A-dione, T-PA, DHT-PA, estrone-PA, and estradiol-2PA were 1000, 40, 20, 10, 20, 10 and 10 pg/mL, respectively. The enzyme ratio of 3β-hydroxysteroid dehydrogenase (3β-HSD), 17β-hydroxysteroid dehydrogenase (17β-HSD), and cytochrome P450-17,20 lyase (CYP17 lyase) were calculated as the similar method in our previous report [7]. Other hormone enzyme activities were determined as:

$$\begin{array}{rl} & \text{Aromatase activity (\%)}\\ & \;=100\times \{(\text{Estrone}+\text{estradiol})/(\text{A-dione}+\text{T})\}\\ & 5\alpha \text{- or }5\beta \text{-Reductase activity}=\text{DHT/T} \end{array}$$

### Dioxin estimation

The levels of the dioxin congeners (7 dioxin and 10 furan congeners) in the maternal breast milk were determined by gas chromatography high-resolution mass spectrometry (JMS-700 MS station Mass Spectrometer; JEOL Ltd., Tokyo, Japan) following a previously reported method [19]. The levels of the PCDDs/Fs were indicated in pg/g of lipid and were converted to toxic equivalents (TEQs) using the World Health Organization 2005 toxic equivalent factors [20].

### Data analysis

The demographic data of the children are presented as mean ± standard deviation for a normal distribution or as median (inter-quantile range) for a non-normal distribution. To improve the normality, the dioxin congener and hormone levels were log10 transformed and are presented as geometric mean ± geometric standard deviation.

All data were checked for distribution by the Shapiro-Wilk test before applying the statistical tests. The mean difference of each indicator between the two areas was calculated using the Student’s t-test or Wilcoxon’s sign rank test. A multiple regression analysis was performed to determine the relation between the demographic indicators or hormones as the dependent variables and mother’s age, mother’s residence, full breastfeeding period of the children, and dioxin congeners in the breast milk as independent variables.

The significance level was set at p < 0.05. All statistical analyses were carried out using JMP 12 (SAS Institute, Cary, NC, USA) and Microsoft Excel 2010 (Microsoft, Redmond, WA, USA).

### Ethics committee

This study was approved by the Medical Ethics Committee (No. 455) of Kanazawa University. The children’s guardians provided consent for their child to participate after being adequately explained the purpose of this study and the procedure of this examination.

## Results

Demographic characteristics of the children divided by boys and girls are indicated in Table 1. As for the girl groups, participants from the hotspot area have significantly lower height, weight, and BMI than in the non-exposure area (p = 0.014, 0.010 and 0.029, respectively), while the head and chest circumferences were not different. Body measurements in boys also did not differ between the two areas.

**Table 1:** Demographic characteristics of children in the hotspot and the non-exposure areas

**Sex**	**parameters**	**Non-exposure area**	**Hotspot**	**p-value**
**Boys**		n = 19	n = 27	
	Height (cm)	132.2 ± 4.7	131.6 ± 5.0	0.672^1)^
	Weight (kg)	27.6 (9.7)	30.8 ± 7.9	0.814^2)^
	BMI (kg/m^2^)	16.9 (3.9)	16.8 (6.4)	0.680^2)^
	Head Circumference (cm)	52.1 ± 1.6	52.6 ± 2.1	0.413^1)^
	Chest Circumference (cm)	62.1 (8.8)	66.4 ± 8.5	0.384^2)^

**Girls**		n = 24	n = 19	
	Height (cm)	130.1 ± 3.9	127.1 ± 5.1	0.014^1)^
	Weight (kg)	26.9 (7.3)	24.7 ± 4.2	0.010^2)^
	BMI (kg/m^2^)	15.8 (3.4)	14.6 (2.7)	0.029^2)^
	Head Circumference (cm)	51.6 ± 1.3	51.3 ± 1.6	0.562^1)^
	Chest Circumference (cm)	60.10 (6.0)	59.4 ± 4.1	0.240^2)^

Data are shown as mean ± standard deviation for the normal distribution data set and median (interquartile range) for the non-normal distribution data set.

BMI: body mass index

^1)^ Student’s *t*-test; ^2)^ Wilcoxon test

The comparison of dioxin congeners in maternal breast milk between the two study areas is shown in Table 2. Significantly higher dioxin and furan congener levels (from 2.0 to 9.8-fold) in the hotspot than those in the non-exposure area were observed, except for 2,3,7,8-TeCDF. Similarly, the TEQs of the total PCDDs, PCDFs, and PCDDs/Fs were 6.5, 4.3 and 11.0 pg/g of lipid 3 times (3.1–3.6) higher in the hotspot area.

**Table 2:** Comparison of dioxin congeners in maternal breast milk between the two study areas

**Dioxin Congeners** **(pg/g of lipid)**	**Non-exposure ** **area** **(n = 43)**	**Hotspot** **(n = 46)**	**Hotpot/Non-exposure ** **ratio**	**p-value**
	
	**GM (GSD)**	**GM (GSD)**		
2,3,7,8-TeCDD	0.3 (2.2)	1.3 (1.8)	3.8	<0.001^2)^
1,2,3,7,8-PeCDD	1.1 (1.6)	3.9 (1.5)	3.5	<0.001^1)^
1,2,3,4,7,8-HxCDD	0.6 (1.5)	1.9 (1.5)	3.2	<0.001^1)^
1,2,3,6,7,8-HxCDD	1.2 (1.6)	6.3 (1.6)	5.2	<0.001^1)^
1,2,3,7,8,9-HxCDD	0.5 (1.7)	2.4 (1.5)	4.5	<0.001^1)^
1,2,3,4,6,7,8-HpCDD	2.3 (1.6)	11.9 (1.6)	5.2	<0.001^2)^
OCDD	10.9 (1.6)	62.9 (1.5)	5.8	<0.001^1)^
2,3,7,8-TeCDF	0.6 (1.6)	0.6 (1.7)	0.9	0.360^2)^
1,2,3,7,8-PeCDF	0.4 (1.7)	1.7 (1.7)	3.9	<0.001^1)^
2,3,4,7,8-PeCDF	2.8 (1.4)	5.6 (1.4)	2.0	<0.001^1)^
1,2,3,4,7,8-HxCDF	1.8 (1.5)	12.7 (1.7)	7.2	<0.001^1)^
1,2,3,6,7,8-HxCDF	1.5 (1.5)	7.6 (1.7)	5.0	<0.001^1)^
1,2,3,7,8,9-HxCDF	0.1 (1.5)	0.3 (1.9)	2.4	<0.001^2)^
2,3,4,6,7,8-HxCDF	0.5 (1.7)	1.4 (1.5)	2.8	<0.001^1)^
1,2,3,4,6,7,8-HpCDF	1.4 (1.8)	13.3 (2.0)	9.8	<0.001^2)^
1,2,3,4,7,8,9-HpCDF	0.2 (1.5)	1.3 (2.0)	7.8	<0.001^1)^
OCDF	0.3 (2.0)	0.9 (2.8)	2.8	<0.001^2)^
TEQ total PCDDs	1.8 (1.6)	6.5 (1.5)	3.6	<0.001^1)^
TEQ total PCDFs	1.4 (1.4)	4.3 (1.5)	3.1	<0.001^1)^
TEQ total PCDDs/Fs	3.2 (1.5)	11.0 (1.4)	3.4	<0.001^1)^

GM: Geometric mean, GSD: Geometric standard deviation

^1)^ Student’s *t*-test; ^2)^ Wilcoxon test

Table 3 shows the comparison of serum steroid hormones and steroid enzyme activities between the two study areas. For both genders, the DHT levels of children from the hotspot (12.6 pg/L in boys and 16.5 pg/L in girls) were remarkably lower than those of the non-sprayed children in both genders (36.1 in boys and 31.5 in girls, respectively). There was no significant difference in A-dione, DHEA, estrone, estradiol and T concentrations between the two areas. Whereas, the cortisol concentrations in the hotspot area were considerably higher than those from the non-exposure area only in girls (p = 0.006). On the other hand, it was observed that the DHT/T ratio in the hotspot declined to half compared to the hotspot area in both boys and girls (p < 0.001). The activity of the Cyp17, 20 lyases, 3β-HSD, 17β-HSD, and aromatase were related to androgen or estrogen synthesis was not significantly different between the two areas.

**Table 3:** Comparison of serum steroid hormones and steroid enzyme activities between the two study areas

**Sex**	**Hormones or enzymes**	**LLOQ** **(pg/injection)**	**Non-exposure area**	**Hotspot**	**p-value**
	
			**GM (GSD)**	**GM (GSD)**	
**Boys**			**n = 19**	**n = 27**	
	Cortisol (ng/mL)	10.0	33.7 (1.8)	34.1 (2.4)	0.428^2)^
	Androstenedione (pg/mL)	0.2	186.4 (2.1)	122.3 (2.2)	0.211^2)^
	DHEA (pg/mL)	2.0	698.0 (1.8)	499.7 (2.8)	0.168^1)^
	Testosterone, T (pg/mL)	0.1	54.0 (1.8)	38.9 (2.0)	0.192^2)^
	Dihydrotestosterone, DHT (pg/mL)	0.1	36.1 (1.8)	12.6 (2.8)	<0.001^1)^
	Estrone (pg/mL)	0.1	2.2 (2.9)	2.6 (2.4)	0.240^2)^
	Estradiol (pg/mL)	0.1	3.1 (3.7)	1.4 (1.9)	0.081^1)^
	CYP-17,20-Lyase Activity (%)		30.0 (2.1)	20.8 (2.3)	0.126^1)^
	17β-HSD Activity (%)		29.0 (1.3)	31.8 (1.3)	0.241^1)^
	3β-HSD Activity (%)		40.1 (1.7)	35.3 (1.9)	0.631^2)^
	Aromatase Activity (%)		2.4 (2.2)	2.7 (1.9)	0.634^1)^
	DHT/T (Ratio)		66.9 (1.4)	32.4 (1.8)	<0.001^2)^

**Girls**			**n = 24**	**n = 19**	
	Cortisol (ng/mL)	10.0	31.3 (1.7)	49.1 (1.6)	0.006^1)^
	Androstenedione (pg/mL)	0.2	171.3 (2.0)	150.2 (2.3)	0.590^1)^
	DHEA (pg/mL)	2.0	758.6 (2.2)	534.9 (2.5)	0.160^2)^
	Testosterone, T (pg/mL)	0.1	49.9 (2.0)	47.2 (1.9)	0.790^2)^
	Dihydrotestosterone, DHT (pg/mL)	0.1	31.5 (1.9)	16.5 (2.2)	0.007^1)^
	Estrone (pg/mL)	0.1	2.1 (2.7)	2.9 (2.8)	0.259^1)^
	Estradiol (pg/mL)	0.1	4.1 (2.9)	3.4 (3.1)	0.444^1)^
	CYP-17,20-Lyase Activity (%)		33.6 (2.3)	16.1 (2.1)	0.002^2)^
	17β-HSD Activity (%)		29.1 (1.3)	31.4 (1.6)	0.981^2)^
	3β-HSD Activity (%)		33.7 (1.7)	41.4 (1.8)	0.120^2)^
	Aromatase Activity (%)		2.9 (2.2)	3.4 (2.0)	0.481^1)^
	DHT/T (Ratio)		63.2 (1.4)	35.1 (1.7)	<0.001^1)^

GM: Geometric mean, GSD: Geometric standard deviation, LLOQ: lower limit of quantification

DHEA: dehydroepiandrosterone, T: testosterone, DHT: dihydrotestosterone

HSD: hydroxysteroid dehydrogenase, CYP-17,20-Lyase: cytochrome P450-17,20-lyase,

3β-HSD Activity (%) = 100 × (androstenedione + testosterone + dihydrotestosterone)/DHEA

17β-HSD Activity (%) = 100 × testosterone / androstenedione

CYP-17,20-Lyase Activity (%) = 100 × (DHEA + androstenedione + testosterone + DHT + estrone + estradiol)/cortisol

Aromatase Activity (%) = 100 × (estrone + estradiol)/(androstenedione + testosterone)

1) Student’s t-test; 2) Wilcoxon test

**Fig. 2:**
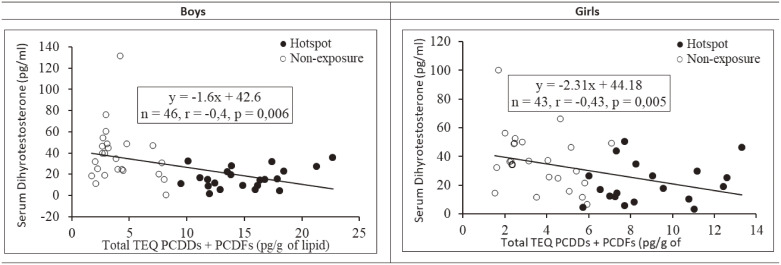
Correlation of TEQs total of PCDDs+PCDFs in maternal breastmilk and DHT level in children’s serum

Figure 2 provides the correlation of TEQs total of PCDDs/Fs in maternal breastmilk and DHT levels in children’s serum. Based on the linear correlation analysis, the DHT levels in boys and girls from the combined area also showed a negative correlation with the TEQ total PCDDs/Fs levels (r = −0.40; p = 0.006 and r = −0.43; p = 0.005).

Table 4 presents a multiple regression correlation of dioxin congeners in maternal breast milk and height, weight, DHT, and estradiol in children. Multiple regression models were applied with the height, weight and DHT as dependent variables and full breastfeeding period of children, mother age, mother residence, and maternal breastmilk dioxin congener levels as the independent variables.

**Table 4:** Multiple regression correlation of dioxin congeners in maternal breastmilk and height, weight, DHT in children

**Sex**	**Dioxin levels in ** **maternal milk**	**Height**			**Weight**			**DHT (pg/mL)**		
		
		**R^2^**	**p**	**β**	**R^2^**	**p**	**β**	**R^2^**	**p**	**β**
**Boys**	2,3,7,8-TeCDD	0.04	0.964	0.007	0.11	0.702	0.058	0.14	0.019	−0.363
	1,2,3,7,8-PeCDD	0.05	0.083	0.063	0.13	0.264	0.167	0.13	0.024	−0.346
	1,2,3,4,7,8-HxCDD	0.04	0.931	0.013	0.12	0.445	0.114	0.15	0.015	−0.371
	1,2,3,6,7,8-HxCDD	0.04	0.916	0.016	0.11	0.568	0.086	0.16	0.010	−0.394
	1,2,3,7,8,9-HxCDD	0.05	0.693	0.061	0.13	0.339	0.143	0.19	0.005	−0.427
	1,2,3,4,6,7,8-HpCDD	0.04	0.975	−0.005	0.11	0.721	0.053	0.23	0.002	−0.467
	OCDD	0.06	0.435	−0.121	0.11	0.800	0.038	0.20	0.003	−0.447
	2,3,7,8-TeCDF	0.05	0.654	−0.071	0.12	0.501	−0.102	0.03	0.371	−0.142
	1,2,3,7,8-PeCDF	0.05	0.654	0.069	0.12	0.450	0.113	0.17	0.008	−0.401
	2,3,4,7,8-PeCDF	0.05	0.731	0.054	0.12	0.451	0.114	0.11	0.035	−0.328
	1,2,3,4,7,8-HxCDF	0.05	0.804	0.038	0.12	0.457	0.111	0.19	0.004	−0.431
	1,2,3,6,7,8-HxCDF	0.05	0.766	0.046	0.12	0.452	0.112	0.20	0.004	−0.435
	1,2,3,7,8,9-HxCDF	0.05	0.676	0.066	0.13	0.326	0.150	0.12	0.032	−0.338
	2,3,4,6,7,8-HxCDF	0.05	0.567	0.088	0.12	0.509	0.098	0.16	0.011	−0.383
	1,2,3,4,6,7,8-HpCDF	0.04	0.861	0.027	0.12	0.485	0.104	0.21	0.003	−0.446
	1,2,3,4,7,8,9-HpCDF	0.04	0.979	−0.004	0.11	0.604	0.078	0.23	0.001	−0.475
	OCDF	0.05	0.741	0.053	0.12	0.402	0.129	0.05	0.176	−0.217
	TEQ total PCDDs	0.05	0.778	0.044	0.12	0.356	0.139	0.15	0.014	−0.375
	TEQ total PCDFs	0.05	0.746	0.050	0.12	0.486	0.104	0.17	0.070	−0.407
	TEQ total PCDDs/Fs	0.05	0.769	0.046	0.12	0.423	0.120	0.16	0.010	−0.393

**Girls**	2,3,7,8-TeCDD	0.17	0.040	−0.323	0.25	0.005	−0.431	0.14	0.034	−0.341
	1,2,3,7,8-PeCDD	0.20	0.020	−0.376	0.23	0.009	−0.420	0.28	0.001	−0.531
	1,2,3,4,7,8-HxCDD	0.20	0.019	−0.381	0.28	0.003	−0.476	0.14	0.036	−0.351
	1,2,3,6,7,8-HxCDD	0.19	0.022	−0.373	0.19	0.029	−0.356	0.20	0.007	−0.441
	1,2,3,7,8,9-HxCDD	0.20	0.017	−0.388	0.20	0.021	−0.373	0.17	0.047	−0.333
	1,2,3,4,6,7,8-HpCDD	0.14	0.099	−0.270	0.15	0.077	−0.288	0.11	0.068	−0.305
	OCDD	0.18	0.029	−0.357	0.20	0.022	−0.372	0.18	0.013	−0.410
	2,3,7,8-TeCDF	0.07	0.771	0.048	0.08	0.894	0.022	0.03	0.885	0.024
	1,2,3,7,8-PeCDF	0.17	0.037	−0.328	0.13	0.125	−0.244	0.05	0.335	−0.159
	2,3,4,7,8-PeCDF	0.10	0.278	−0.174	0.14	0.109	−0.253	0.11	0.077	−0.286
	1,2,3,4,7,8-HxCDF	0.18	0.033	−0.349	0.18	0.033	−0.349	0.12	0.063	−0.314
	1,2,3,6,7,8-HxCDF	0.17	0.039	−0.339	0.19	0.026	−0.364	0.10	0.104	0.276
	1,2,3,7,8,9-HxCDF	0.10	0.357	−0.148	0.03	0.821	−0.038	0.11	0.260	−0.181
	2,3,4,6,7,8-HxCDF	0.13	0.128	−0.245	0.18	0.038	−0.328	0.07	0.189	−0.217
	1,2,3,4,6,7,8-HpCDF	0.18	0.028	−0.349	0.17	0.047	−0.316	0.12	0.050	−0.320
	1,2,3,4,7,8,9-HpCDF	0.20	0.017	−0.387	0.18	0.035	−0.342	0.09	0.136	−0.252
	OCDF	0.16	0.055	−0.307	0.12	0.187	−0.213	0.03	0.937	0.013
	TEQ total PCDDs	0.20	0.018	−0.376	0.25	0.006	−0.432	0.23	0.003	−0.470
	TEQ total PCDFs	0.15	0.074	−0.290	0.18	0.040	−0.330	0.10	0.085	−0.286
	TEQ total PCDDs/Fs	0.19	0.025	−0.359	0.23	0.010	−0.406	0.18	0.012	−0.410

β: standardized coefficients, p: p-value, R^2^: coefficient of determination, DHT: dihydrotestosterone.

Dependent variates are height, weight and DHT.

Independent variates are mother age, residence, full breastfeeding period and dioxin congener.

Besides that, there was a sex-specific difference in the correlation of maternal dioxin levels and parameters in children. For the body measurements, the height and weight showed inverse correlations with almost all the dioxins (except for 1,2,3,4,6,7,8-HpCDD), some furan congeners (1,2,3,4,7,8-HxCDF; 1,2,3,6,7,8-HxCDF; 1,2,3,4,6,7,8-HpCDF; 1,2,3,4,7,8,9-HpCDF) and the TEQ total dioxin (except for TEQ total PCDFs for height) only in the girls.

On the other hand, the inverse correlations with dioxin congeners were observed for DHT levels in boys (except for 2,3,7,8-TeCDF, OCDF and TEQ total PCDFs). For girls, there were negative correlations between DHT and PCDD congeners (except for 1,2,3,4,6,7,8-HpCDD); 1,2,3,4,6,7,8-HpCDF, TEQ total PCDDs and TEQ total PCDDs/Fs).

## Discussion

We have continued follow-up studies on the effects of maternal breast dioxin exposure (hotspot) on mother-child pairs [6–8]. In this study, we at the first time showed that the serum DHT levels of 9-year-old children from the hotspot were significantly lower than those of the non-exposure children and also showed significant inverse correlations with almost the PCDDs/Fs congeners and total TEQs dioxin in both genders (Table 3 and Fig. 2). Furthermore, we analyzed so-called “female sex hormones” such as estrone and estradiol in this 9-year-old study at the first time, too. However, their levels did not differ between two areas.

As published, DHEA levels declined from 3 to 5-year-old children from the hotspot in both genders, then rapidly increased and recovered to levels in the non-sprayed area in girls or even significantly higher in boys at 7-year-old children [6–8]. While T levels in boys and girls from the hotspot were still significantly lower at both 5 and 7 age [7, 8]. And now, they recovered and showed no difference between the two areas in 9-year-old children. The cortisol levels of girls in the hotspot area were significantly higher than those from the non-exposure area. The high cortisol levels are similar to their mother’s pattern [21]. The other estimated steroid levels were not significantly different between the two areas.

DHT has a strong androgenic effect and plays an important role in the development of organogenesis [22]. The DHT concentration is important for considering the health effects of dioxins and their mechanisms, especially since it is known to be involved in sexual tissue differentiation. O’Shaughnessy et al proposed that T and DHT have different androgenic effects on the development of the male reproductive organs [23]. T reportedly acted on penile development and spermatogenesis, and DHT acted on the external reproductive organs [24]. Therefore, the suppressive androgen action of dioxin exposure is known to suppress the development of the penis, suppress the secretion of semen, and make vulvar feminization. The dioxin attenuates both the T and DHT androgen action through steroid hormone levels.

Next, we focused on the mechanism of DHT levels in children from the non-exposure and hotspot areas. The 5α-reductase activity, in this study, was indirectly calculated by the ratio of DHT/T, which reflects the conversation of DHT from T. The ratio of the DHT/T circulating concentration in children from the non-exposure group was about 60% (as shown in Table 3), which was considerably higher than that of 10% in the adults described by Sun et al and Caron et al [4, 25]. This showed that the DHT/T ratio and DHT level significantly varied with age, and our results were consistent with the findings of other researchers [26].

The 5α-reductase type 1 and type 2 are known to be enzymes involved in DHT production. It has been reported that type 2, mainly expressed in the prostate and reproductive organs, acts on the DHT production from T, and affects the development of the urogenital system and the etiology of prostatic hyperplasia [27]. Inactivating mutations in type 2 lead to disorders of sexual development. On the other hand, the 5α-reductase type 1 is present in many organs including the liver, and the enzyme acts on the C-19 steroids of T and A-dione and the C-21 steroids of progesterone or cortisol [28]. DHT is generally thought to be produced from T by 5α-reductase type 2 in humans [27]. The difference in the DHT/T ratio between the hotspot and non-sprayed areas is presumed to be related to the amount of the 5α-reductase type 1 and 2 activities or suggests the existence of other DHT production pathways.

Based on the study of Wilson et al and Auchus et al, a system that produces DHT from the so-called progesterone route via 5α-pregnane-3,20-dione as an intermediate was discovered in kangaroos [29, 30]. This system is called the backdoor pathway by Miller and Auchus, R.J. [31]. Japanese researchers confirmed the presence of the pathways in a clinical study [32]. Reish et al provided in vitro (adrenal, gonad, genital skin) and vivo (urine) evidence for the existence of the backdoor pathway for the biosynthesis of the DHT during human development (male and female) [33]. Its presence has been demonstrated in 5α-reductase type 1 knockout mice and has been noted to be involved in vulvar feminization [34, 35].

On the other hand, we also observed the smaller weight and height in 9-year-old girls from the hotspot area than those from the non-exposure area as previously shown in the 7-year-old girls [8]. Lamb et al reported that maternal PCB levels were associated with reduced weight through 17 years of age among girls only [36]. However, whether this effect is related to the dioxin /PCB levels or hormone disruption is unclear.

More recently, Manh et al have reported that the serum dioxin levels of children living in the hotspot were over 3-fold higher than those in the non-exposure area, similar to the one’s levels in mothers’ breast milk (Table 2) [37]. Therefore, the dioxin burden in children associated with prenatal dioxin and intake of maternal breast milk produces androgen disturbance until 9 years old. Vandenberg et al. suggested that endocrine disruptions can have effects at low doses that are not predicted by the effects of a higher dose [38].

In this study, we showed that dioxin could lead to the risk of health issues due to steroid hormone disruptions in a child’s developmental process. DHT is a more potent androgen than T, however, the DHT levels are only about 10% of the T in a normal adult. DHT is an important diagnostic parameter for infants and boys to evaluate the disorders of sex development because it is the key androgen for the formation of the normal fetal genitalized male. Therefore, these facts led us to the analysis of a few DHT using the LC-MS/MS levels in children.

Several limitations should be considered in this study. First, estradiol estimation must be developed with the more highly sensitive method by the LC-MS/MS because of its low concentration in children. In fact, estradiol in children was not detected in 15 samples from the non-exposure area and 20 samples from the hotspot in this study. Therefore, we did not find a significant difference in the estradiol levels between the two areas in both genders. Second, if the catalytic enzyme activities in androgen biosynthesis were measured directly rather than calculated through the ratio between hormones, it would give clear suggestions of the mechanism of dioxin causing hormone disruption. The small sample size of the study, finally, also needs to be mentioned.

## Conclusion

We have clarified the transition of dioxin-dependent androgen disruption in Vietnamese children versus age, including decreases in DHEA and subsequent in T. The most important finding in the 9-year-old children is a marked decrease in the most potent androgen, DHT level in the dioxin hotspot. The mechanism of this disruption is unknown. Continuing investigation of sex hormone disorders and reproductive characteristics by dioxin exposure when children are at puberty is necessary to have timely and appropriate intervention measures.

## Abbreviations

3β-HSD: 3β-hydroxysteroid dehydrogenase
17β-HSD: 17β-hydroxysteroid dehydrogenase
A-dione: Androstenedione
BMI: Body Mass Index
CYP17 lyase: Cytochrome P450-17,20 lyase
DHEA: Dehydroepiandrosterone
DHT: Dihydrotestosterone
LS-MS/MS: Liquid chromatography-tandem mass spectrometry
T: Testosterone
PCDDs: Polychlorinated dibenzo-p-dioxins
PCDFs: Polychlorinated dibenzo-furans
TEQs: Toxic equivalents

## Declarations

### Availability of data and materials

Data available on request from authors. However, the individual information of the participants in this study is not publicly available due to the discontent with the disclosure from the participants.
